# High burden of ESBL and carbapenemase-producing gram-negative bacteria in bloodstream infection patients at a tertiary care hospital in Addis Ababa, Ethiopia

**DOI:** 10.1371/journal.pone.0287453

**Published:** 2023-06-27

**Authors:** Daniel Beshah, Adey Feleke Desta, Gurja Belay Woldemichael, Esmael Besufikad Belachew, Solomon Gizaw Derese, Tizazu Zenebe Zelelie, Zelalem Desalegn, Tesfaye Sisay Tessema, Solomon Gebreselasie, Tamrat Abebe

**Affiliations:** 1 Microbial Cellular and Molecular Biology Department, College of Natural and Computational Sciences, Addis Ababa University, Addis Ababa, Ethiopia; 2 Department of Diagnostic Laboratory, Tikur Anbessa Specialized Hospital, College of Health Sciences, Addis Ababa University, Addis Ababa, Ethiopia; 3 Department of Biology, College of Natural and Computational Sciences, Mizan-Tepi University, Tepi, Ethiopia; 4 Department of Microbiology, Immunology, and Parasitology, School of Medicine, College of Health Sciences, Addis Ababa University, Addis Ababa, Ethiopia; 5 Department of Medical Laboratory Science, Debre Berhan University, Debre Berhan, Ethiopia; 6 Institute of Biotechnology, Addis Ababa University, Addis Ababa, Ethiopia; Wollo University, ETHIOPIA

## Abstract

**Background:**

Bloodstream infection due to beta-lactamase and carbapenemase-producing gram-negative bacteria poses a substantial challenge to the effectiveness of antimicrobial treatments. Therefore, this study aimed to investigate the magnitude of beta-lactamase, carbapenemase-producing gram-negative bacteria, and associated risk factors of bloodstream infections in patients at a tertiary care hospital, in Addis Ababa, Ethiopia.

**Methods:**

An institutional-based cross-sectional study was conducted with convenience sampling techniques from September 2018 to March 2019. Blood cultures were analyzed from 1486 bloodstream infection suspected patients across all age groups. The blood sample was collected using two BacT/ALERT blood culture bottles for each patient. Gram stain, colony characteristics, and conventional biochemical tests were used to classify the gram-negative bacteria at the species level. Antimicrobial susceptibility testing was carried out to screen beta-lactam and carbapenem drug-resistant bacteria. The E-test was conducted for extended-spectrum-beta-lactamase and AmpC-beta-lactamase-producers. A modified and EDTA-modified carbapenem inactivation method was conducted for carbapenemase and metallo-beta-lactamases producers. Data collected using structured questionnaires and medical records were reviewed, encoded, and cleaned using EpiData V3.1. software. The cleaned data were exported and analyzed using SPSS version 24 software. Descriptive statistics and multivariate logistic registration models were used to describe and assess factors associated with acquiring drug-resistant bacteria infection. A p-value <0.05 was considered statistically significant.

**Result:**

Among 1486 samples, 231 gram-negative bacteria were identified; of these, 195(84.4%) produce drug-hydrolyzing enzymes, and 31(13.4%) produce more than one drug-hydrolyzing enzyme. We found 54.0% and 25.7% of the gram-negative bacteria to be extended-spectrum-beta-lactamase and carbapenemase-producing, respectively. The extended-spectrum-beta-lactamase plus AmpC-beta-lactamase-producing bacteria account for 6.9%. Among the different isolates *Klebsiella pneumonia* 83(36.7%) was the highest drug-hydrolyzing enzyme-producing bacteria. *Acinetobacter spp* 25(53.2%) was the most carbapenemase producer. Extended-spectrum-beta-lactamase and carbapenemase-producing bacteria were high in this study. A significant association between age groups and extended-spectrum-beta-lactamase producer bacterial infection was seen, with a high prevalence in neonates (p = <0.001). Carbapenemase showed a significant association with patients admitted to the intensive care unit (p = 0.008), general surgery (p = 0.001), and surgical intensive care unit (p = 0.007) departments. Delivery of neonates by caesarean section, and insertion of medical instruments into the body were exposing factors for carbapenem-resistant bacterial infection. Chronic illnesses were associated with an extended-spectrum-beta-lactamase-producing bacterial infection. *Klebsiella pneumonia and Acinetobacter species* showed the greatest rates of extensively drug-resistant (37.3%) and pan-drug-resistance (76.5%), respectively. According to the results of this study, the pan-drug-resistance prevalence was found to be alarming.

**Conclusion:**

Gram-negative bacteria were the main pathogens responsible for drug-resistant bloodstream infections. A high percentage of extended-spectrum-beta-lactamase and carbapenemase-producer bacteria were found in this study. Neonates were more susceptible to extended-spectrum-beta-lactamase and AmpC-beta-lactamase-producer bacteria. Patients in general surgery, caesarean section delivery, and intensive care unit were more susceptible to carbapenemase-producer bacteria. The suction machines, intravenous lines, and drainage tubes play an important role in the transmission of carbapenemase and metallo-beta-lactamase-producing bacteria. The hospital management and other stakeholders should work on infection prevention protocol implementation. Moreover, special attention should be given to all types of *Klebsiella pneumoniae* and pan-drug resistance *Acinetobacter spp* transmission dynamics, drug resistance genes, and virulence factors.

## Introduction

Gram-negative bacteria (GNB) are one of the major global health concerns due to beta-lactamase and carbapenemase production, which leads to antimicrobial resistance (AMR) [1, 2]. It has become an important cause of morbidity and mortality worldwide [3, 4]. In 2050, multidrug-resistant bacteria are expected to be the cause of 10 million annual deaths [5]. Over 19% of nosocomial infections are caused by extended-spectrum beta-lactamase (ESBL) enzyme-producing bacteria. The ESBL-producing bacteria were also expected to cause a 57% higher mortality rate among bloodstream infections (BSI) than a strain that doesn’t produce ESBL [6]. Production of ESBL and carbapenemase are causes of drug resistance, particularly in GNB, and these pauses serious challenges to the effectiveness of contemporary antibiotic treatment [6, 7]. The ESBL enzyme can hydrolyze penicillin, monobactam, and third-generation cephalosporins. AmpC beta-lactamase (AmpC-BL) can also break down penicillins, third-generation cephalosporins, and cephamycin, while carbapenemase and metallo-beta-lactamase (MBL) can break down carbapenems drugs [6–9]. Recently, it has been observed that the multi-drug resistance (MDR) problem has shifted from gram-positive bacteria to GNB, which in turn worsens the problem by the dearth of new antimicrobial drugs having an effect against them [10]. The GNB, particularly ESBL and carbapenemase-producing *Klebsiella pneumoniae*, *Escherichia coli*, *Acinetobacter baumannii*, *Proteus mirabilis*, and *Pseudomonas aeruginosa*, showed increasing drug-resistance mechanism [4]. Research conducted in an Ethiopian context showed that there is a significant association between death and AMR in patients with BSI [11]. Similar research had also reported a prevalence of ESBL and AmpC-BL in GNB to be 38.8% and 2.4% [12], 67% and 2% [13], 51% and 25% [14], and in *Enterobacteriaceae* specifically, it *was* reported to be 78.6% and 12.1% [15], respectively. Another research conducted on GNB in an Ethiopian context has also shown a magnitude of 94.5% of the isolates to be MDR of which 8.8% and 0.8% were due to extensively drug-resistance and pan-drug resistant, respectively [13]. However, most of these studies did not address the magnitude of ESBL and carbapenem-producing GNB and the associated factors among all age groups of BSI in tertiary hospitals. Therefore, the objective of this study was to address the identified gaps. Mainly the study aimed to show the magnitude of ESBL, AmpC-BL, MBL, and Carbapenemase-producing GNB and associated risk factors among bloodstream infections patients in Tikur Anbessa Specialized Hospital (TASH), Addis Ababa, Ethiopia.

## Materials and methods

### Study design, study area, study population, and sample size

An institutional-based cross-sectional study was conducted from September 2018 to Mar 2019 in TASH which is a tertiary hospital located in Addis Ababa having over 700 beds, 201 doctors, 627 nurses, more than 115 other health professionals, and 1300 administrative staff that are dedicated to providing healthcare services [16]. The study population consists of all patients who have visited TASH for medical care during the study period. Convenience sampling techniques were deployed to all age groups of BSI-suspected patients who agreed to offer a blood sample and sign a consent form to participate in the study. Patients that were not willing to participate in the study and had been on antibiotic treatment within ten days were excluded. A total of 1486 patients were enrolled in this study and the sample size calculation was based on a single sample size estimation [17, 18] with a prevalence of 32.8% taken from the study conducted in Addis Ababa, Ethiopia [19]. A sample size (n) calculation with *n* = *Z*^2^*p* (1 –*p*)/*d*^2^, and *n* = 1.96^2^0.328(1–0.328)/(0.025)^2^ = 1354.80 with 10% contingency gives the size to be = 1490. To increase the accuracy of the result a margin of error (d) = 0.02 was used to maximize the sample size. Z stands for Z score with a level of confidence of 95%, which is a conventional value with a Z value of 1.96.

### Data collection

Patient demographic data and clinical information were collected using a standardized questionnaire and review of patient medical records by trained nurses. Blood samples were collected by qualified professional nurses and laboratory technologists who had prior experience in collecting research data and blood samples. All data and sample collectors were trained by the principal investigator on the detailed instructions of using prepared standardized questionnaires, blood sample collection procedures, and sample transportation to the laboratory. Follow-up of microbial growth in the blood culture bottle and the downstream steps were accomplished by the recruited professional microbiologists and the principal investigator in the laboratory.

### Sample collection

All age groups of hospitalized patients with a bloodstream infection who signed an informed consent form and were willing to participate in the study were included. The conditions of bloodstream infection were defined by the 1991 consensus conference, which was later updated in 2001 [20, 21]. We classified eligible patients as bloodstream infection cases based on the attending physician’s judgment. Blood samples were collected aseptically from bloodstream infection suspected patients who meet the criteria set in this study. The vein puncture site was disinfected with 70% alcohol and 2% tincture of iodine before blood collection. Approximately 1–4 ml of blood from pediatrics (less than 12 years) and 5–10 ml of blood from adults (greater than 12 years) were taken and inoculated into a ready-made BacT/ALERT blood culture bottle [22]. Two aerobic BacT/Alert FA Plus and BacT/Alert PF Plus bottles were used for blood culture, among adults and pediatrics, respectively. Similar growth in both bottles was considered positive. Blood culture bottles were incubated with an automatic BacT/ALERT® 3D machine at 37°C of 5% CO_2_ gas concentration which was inspected daily for the sign of bacterial growth for up to 5 days [23, 24].

### Bacteria identification

Blood samples that were turbid before five days and blood samples that were not turbid at five days were sub-cultured on blood agar (Oxoid, England) (5% sheep blood plates), MacConkey agar (BD, USA), and chocolate agar (Oxoid, England), at 37°C for 24 hours. The McConkey plates were incubated in aerobic conditions, while chocolate and blood agar plates were incubated in microaerophilic conditions (containing 5% CO_2_) [25]. Bacterial pathogens were identified using CLSI 2017 standard bacteriological procedures [26]. Hence a positive culture from blood samples was characterized by their colony characteristics and gram stain. The GNB bacteria were further classified at the species level by using conventional biochemical tests including indole, urea agar, triple sugar iron agar, citrate utilization, mannitol, malonate, motility, lysine decarboxylase, and oxidase test [27].

### Antimicrobial susceptibility testing

Antimicrobial susceptibility tests (AST) were performed by using disk diffusion and the susceptibility range was determined based on CLSI guidelines [26]. With that, each isolate was spread onto a Mueller–Hinton agar plate. The antimicrobials for disc diffusion testing was obtained from BD and Oxoid for GNB with the following concentrations: for GNB Amikacin (AN) (30 μg), Amoxicillin + clavulanic acid (AMC) (20/10μg), Ampicillin–Sulbactam (SAM) (10/10 μg), Ampicillin (AM) (10 μg), Azithromycin (ATM) (30 μg), Cefepime (FEP) (30 μg), Cefotaxime–clavulanate (CTL) (30/20μg), Cefotaxime (CTX) (30 μg), Cefotetan (CTT) (30 μg), Cefoxitin (FOX) (30 μg), Ceftaroline (CPT) (30 μg), Ceftazidime–clavulanate (CAL) (30/10 μg), Ceftazidime (CAZ) (30 μg), Ceftriaxone (CRO) (30 μg), Cefuroxime (CXM) (30 μg), Chloramphenicol (C) (30 μg), Ciprofloxacin (CIP) (5 μg), Dorsapenem (DOR) (10 μg), Doxycycline (DOX) (30 μg), Ertapenem (ETP) (10 μg), Gentamicin (GM) (10 μg), Imipenem (IMP) (10 μg), Levofloxacin (LEV) (5 μg), Meropenem (MEM) (10 μg), Piperacillin–Tazobactam (TZP) (100/10μg), Tetracycline (TET) (30 μg), Tobramycin (NN) (10 μg), and Trimethoprim-Sulfamethoxazole (SXT) 1(.25/23.75μg) [26, 28].

### ESBL primary screening

Initial screening for ESBL was conducted by the diameter zone of inhibition produced by Ceftazidime (30μg), Ceftriaxone (30μg), and Cefotaxime (30μg) which is found to be within the clinical and laboratory standard institute (CLSI) screening criteria. These breakpoints indicative of ESBL production were CAZ ≤ 22mm, CRO ≤ 25 mm, and CTX ≤ 27mm. Phenotypic detection of ESBL production was confirmed by a double-disk synergy test and combined disk test using an E-test according to EUCAST and CLSI guidelines respectively [26, 29].

#### Double disk synergy test (DDST) for ESBL detection

The organism to be tested were inoculated onto a Mueller–Hinton agar plate. The antibiotic disks that deployed for this purpose were Ceftriaxone (30 μg), Cefotaxime (30 μg), Ceftazidime (30 μg), Aztreonam (30μg), and Amoxicillin/ Clavulanic acid (20/10 μg). The four antibiotics were then placed at distances of 20 mm (edge to edge) from the Amoxicillin/Clavulanic acid disk, which was placed in the middle of the plate. After 24-hrs of incubation, the occurrence of an enhanced zone of inhibition between either of the cephalosporin antibiotics and the Amoxicillin/Clavulanic acid disk was considered ESBL positive [26]. The primary AST screening has integrated the DDST and later on the confirmatory E-test for ESBL and AmpC-BL identification.

#### E-test for both ESBL and AmpC beta-lactamase detection

The E-test was conducted for ESBL detection using Ceftazidime (CAZ) 0.5–32 μg/ml alone with Ceftazidime+ Clavulanic acid (CAL) 0.064–4 μg/ml combination, and Cefotaxime (CTX) 0.25–16 μg/ml alone with Cefotaxime+ Clavulanic acid (CTL) 0.016–1.0 μg/ml combination strip were used for phenotypic confirmation. For detection of ESBL, CTX ≥ 0.5 and CTX/CTL ratio ≥ 8 or CAZ ≥ 1 and CAZ/CAL ratio ≥ 8 were considered as positive, whereas CTX < 0.5 or CTX/CTL ratio < 8 and CAZ < 1 or CAZ/CAL ratio < 8 was considered as negative. The AmpC-BL detection, hence, was done by using cefotetan 0.60–32μg/ml alone with cefotetan-cloxacillin 0.5–32μg/ml combination. The ratio of CTT/CXT ≥8 indicates the production of an AmpC BL [30–32].

### Carbapenem-resistant detection

The presence of Carbapenemase detection was implemented by using the modified Carbapenemase inactivation method (mCIM) and EDTA-modified Carbapenemase inactivation method (eCIM), except for the case of *Acinetobacter spices*. This was conducted for all the Carbapenem-resistant or intermediate with either Imipenem, Meropenem, Dorsapenem, or Ertapenem except for none fermenter GNB where Ertapenem was not used. To perform the mCIM, a suspension was made by taking 1μl loopful of bacteria from an overnight blood agar plate and then adding it to a 2 ml trypticase soy broth. Subsequently, Meropenem (10 μg) disc was immersed in the suspension and incubated for 4 hours ± 15 minutes at 37°C. After the incubation, the disc was removed from the suspension using 10 μl inoculation loops and placed on a Mueller-Hinton agar plate inoculated with a susceptible *E*. *coli* indicator strain (ATCC 29522). Then, the results reading was recorded after 18–24 hours of incubation at 37°C. Consequently, for the eCIM case taking 1μl loopful of bacteria from an overnight blood agar plate and then adding it to 2 ml having 5 molar EDTA concentration trypticase soy broth. Then continued the mCIT test procedure for the identification of MBL production. For mCIM zone of inhibition of 6 to 15 mm around a disk and a zone of inhibition of 16 to 18 mm around a disk including the presence of colonies in the zone of inhibition was considered as positive. A zone of inhibition with a distance of 19 mm or greater around a disk was considered negative. A negative result suggests that the isolate is resistant to carbapenems by a mechanism other than the production of carbapenemase. If mCIT shows carbapenem production and an increase diameter of eCIT greater or equal to 5mm then mCIT was considered as MBL positive [26, 29, 33, 34].

### Determination of MDR, XDR, and PDR isolates

Multidrug resistance (MDR), extensively drug-resistant (XDR), and pan-drug-resistant (PDR isolates were categorized by using Magiorakos *et al*. standard definitions for drug resistance bacteria [35].

### Quality control

Standard operating procedures were strictly followed to verify that quality control parameters were fulfilled. Visual inspections of cracks in media, unequal fill, hemolysis, evidence of freezing, bubbles, and contamination were performed. According to standard operating procedures, blood samples were drawn and then transferred to blood culture broth and immediately transported to the microbiology laboratory [22, 24]. Quality control was performed to check the quality of the media. The resistance and susceptibility were interpreted according to the CLSI laboratory standards. *Escherichia coli* (ATCC 25922), *Staphylococcus aureus* (ATCC 25923), and *Pseudomonas aeruginosa* (ATCC 27853) were used as reference strains for culture and susceptibility tests [26]. *Klebsiella pneumoniae* ATCC® 700603 and *E*. *coli* ATCC® 35218 were used for positive control and negative control for E-test. *Klebsiella pneumoniae* ATCC® 700603 (ESBL positive) was used as a negative control and *Klebsiella pneumoniae* ATCC® BAA-1144 was used as a positive control for E-test AmpC detection. *Klebsiella pneumoniae* ATCC® BAA-1705™ and *Klebsiella pneumoniae* ATCC® BAA-1706 were used as positive and negative controls for the carbapenemase test respectively [31, 32]. These strains were obtained from the TASH.

### Statistical analysis and interpretation

Data collected using a structured questionnaire was encoded and cleaned using EpiData V3.1. software. The cleaned data were exported and analyzed using SPSS version 24 software. The collected quantitative data were analyzed using simple descriptive statistics i.e., percentages, frequency, mean, and standard deviation moreover, the data were analyzed using univariate, and multivariate logistic registration models to assess dependent and independent variables association factors. A p-value of less than 0.05 was considered a statistically significant association. Finally, the results were tabulated hereunder.

### Ethics approval and consent to participate

The study was approved by “The College of Natural and Computational Sciences Institutional Research Ethics Review Board (CNS-IRB) on 30/03/2018 with minutes of meeting reference no IRB/032/2018 at Addis Ababa University”. Permission was also obtained from the College of Health Sciences Institutional Review Board (IRB). This study procedure has fulfilled the requirements set by the Declaration of Helsinki [36]. For participants whose age was below 18 years, data were collected with the assent (12–18) and consent of the patient’s legal guardians. However, in the cases of above 18 years of age, we have collected consent from the patients themselves. The purpose and procedures of the study were explained to the participants’ parents, and/or guardians during the study period. Those patients who were given informed consent were selected and enrolled as the study participants and patient results were communicated to the attending physicians.

## Results

### Culture result

Among 1486 BSI-suspected patient samples, 28.1% were culture positive. Most of the bacteria 233 (54.2%) isolated were GNB. The detail of the bacteria identified and the antimicrobial susceptibility pattern of the isolates was described in our published article [37]. After excluding one *Corynebacterium gleum* and one *Neisseria meningitidis*, a total of 231(54.1%) GNB bacteria were further analyzed for determining their drug resistance mechanisms. Among the 231 GNBs, 84.4% of the isolates were positive for one or two types of drug hydrolyzing enzyme. Out of the total 231 identified GNB, *Klebsiella pneumoniae* (32.5%), *Acinetobacter spp* (20.4%), and *Escherichia coli* (16.5%), were the predominant isolates. A total of 71% of bacteria produced one of the drug hydrolyzing enzymes. Of these 13.4% GNB produced a double drug hydrolyzing enzyme, with ESBL + AmpC-BL producers accounting for 6.9%, ESBL + carbapenemase producers for 5.2%, and ESBL + MBL producers for 1.3%. Of the GNB that produced double drug hydrolyzing enzymes *Klebsiella pneumoniae* and *Acinetobacter spp* account for 35.5% and 25.8%, respectively as shown in Table 1.

**Table 1:** Prevalence of GNB that produces drug hydrolyzing enzymes of ESBL or AmpC-BL or MBL or carbapenemase among BSI-suspected patients.

Gram-negative bacteria	One or two DHE positive N (%)	GNB Having drug hydrolyzing enzymes			Total N
		Singe positive N (%)	Double positive N (%)	Negative N (%)	
*K*. *pneumoniae*	72(96%)	61(81.3%)	11(14.7%)	3(4%)	75
*Acinetobacter spp*	41(87.2%)	33(70.2%)	8(17%)	6(12.8%)	47
*E*. *coli*	28(73.7%)	25(65.8%)	3(7.9%)	10(26.3%)	38
*K*. *oxytoca*	25(92.6%)	20(74.1%)	5(18.5%)	2(7.4%)	27
*E*. *coli(A-D)*	9(81.8%)	8(72.7%)	1(9.1%)	2(18.2%)	11
*C*. *diversus*	2(40%)	2(40%)	0(0%)	3(60%)	5
*S*. *marcescens*	4(100%)	3(75%)	1(25%)	0(0%)	4
*Pseudomonas spp*	4(80%)	4(80%)	0(0%)	1(20%)	5
*K*. *ozaenae*	1(33.3%)	0(0%)	1(33.3%)	2(66.7%)	3
*K*. *rhinoscleroma*	2(50%)	2(50%)	0(0%)	2(50%)	4
*P*. *mirabilis*	1(50%)	0(0%)	1(50%)	1(50%)	2
*E*. *cloacae*	1(50%)	1(50%)	0(0%)	1(50%)	2
*E*. *agglomerans*	1(50%)	1(50%)	0(0%)	1(50%)	2
*M*. *morganii*	1(50%)	1(50%)	0(0%)	1(50%)	2
*P*. *rettgeri*	2(100%)	2(100%)	0(0%)	0(0%)	2
*E*. *aerogenes*	1(100%)	1(100%)	0(0%)	0(0%)	1
*S*. *typhi*	0(0%)	0(0%)	0(0%)	1(100%)	1
**Total**	195(84.4%)	164(71%)	31(13.4%)	36(15.6%)	**231**

DHE; Drug hydrolyzing enzyme, GNB; Gram-negative bacteria.

### Carbapenemase, MBL, ESBL, and AmpC-BL, producer gram-negative bacteria

Out of the total 231 gram-negative bacteria ESBL, Carbapenemase, AmpC-BL, and MBL producers accounted for 52.8%, 25.1%, 14.3%, and 5.6%, respectively. Most *Klebsiella pneumoniae* (77.3%) and *Klebsiella oxytoca* (77.8%) were ESBL producers likewise, most *Acinetobacter spp* (53.2%) were carbapenemase producers (Table 2).

**Table 2:** Prevalence of drug hydrolyzing enzyme production in GNB among BSI patients.

Gram-negative bacteria	Total number of strains	Drug hydrolyzing enzymes identified			
		ESBL N (%)	AmpC-BL N (%)	Carbapenemase N (%)	MBL N (%)
*K*. *pneumoniae*	75	58(77.3%)	6(8%)	15(20%)	4(5.3%)
*Acinetobacter spp*	47	10(21.3%)	9(19.1%)	25(53.2%)	5(10.6%)
*E*. *coli*	38	17(44.7%)	8(21.1%)	4(10.5%)	2(5.3%)
*K*. *oxytoca*	27	21(77.8%)	4(14.8%)	5(18.5%)	0(0%)
*E*. *coli(A-D)*	11	7(63.6%)	1(9.1%)	1(9.1%)	1(9.1%)
*C*. *diversus*	5	2(40%)	0(0%)	0(0%)	0(0%)
*S*. *marcescens*	4	2(50%)	1(25.0%)	2(50%)	0(0%)
*Pseudomonas spp*	5	0(0%)	2(40%)	2(40%)	0(0%)
*K*. *ozaenae*	3	1(33.3%)	1(33.3%)	0(0%)	0(0%)
*K*. *rhinoscleroma*	4	0(0%)	0(0%)	1(25.0%)	1(25.0%)
*P*. *mirabilis*	2	1(50%)	0(0%)	1(50%)	0(0%)
*E*. *cloacae*	2	0(0%)	0(0%)	1(50%)	0(0%)
*E*. *agglomerans*	2	1(50%)	0(0%)	0(0%)	0(0%)
*M*. *morganii*	2	0(0%)	0(0%)	1(50%)	0(0%)
*P*. *rettgeri*	2	2(100%)	0(0%)	0(0%)	0(0%)
*E*. *aerogenes*	1	0(0%)	1(100%)	0(0%)	0(0%)
*S*. *typhi*	1	0(0%)	0(0%)	0(0%)	0(0%)
Total	**231**	122(52.8%)	33(14.3%)	58(25.1%)	13(5.6%)

ESBL: Extended-spectrum beta-lactamase; AmpC-BL: AmpC beta-lactamase; MBL: Metallo beta-lactamase

### Factor associated with carbapenemase and MBL-producing gram-negative bacterial infection

From the 417(28.06%) culture-positive patients, 224(53.72%) were infected with GNB. Of these, 189(84.4%) isolated s bacteria showed the production of one or two drug-hydrolyzing enzymes (S1 Table). However, in cases of polymicrobial growth and double drug hydrolyzing enzyme production, the higher-grade drug hydrolyzing enzyme production was considered for the associated factor analysis.

The multivariate regression analysis of the study revealed significant associations between carbapenemase and MBL producers of GNB and the departments where the patient was admitted. Among the departments where the patients were admitted, general surgery (p = 0.001), surgical intensive care unit (SICU) (p = 0.007), and intensive care unit (ICU) (p = 0.008) were sites where patients will acquire almost five times and two times more likely to acquire carbapenemase and MBLs producer bacteria than those who were admitted in other wards. A significant association to acquire infection with carbapenemase and MBLs producing GNB and care that involves the insertion of medical instruments had been observed (p = 0.001). Moreover, patients in whom medical instruments were inserted during medical care were almost four times more likely to acquire infection with carbapenemase and MBLs-producing bacteria than those who don’t require instrumentation. The use of intravenous lines (p = 0.045), drainage tubes (p = 0.032), and the use of suction machines (p = 0.040) were also associated with carbapenemase and MBL-producing GNB infection.

Chronic wound infection on the skin (p = 0.030), and urinary tract infection (UTI) (p = 0.007), had a significant association with carbapenemase and MBL producers GNB infection (Table 3). Patients with wound infection on the skin and ureteral tract infection were almost 4 times more likely to acquire carbapenemase and MBLs producer bacteria than those not have wound and ureteral tract infected patients (Table 2).

**Table 3:** Bivariate and multivariate logistic regression analysis of demographic and other parameters, taken as predictive variables for acquiring infections with carbapenemase and MBL-producing bacteria.

Variables	Carbapenemase and MBL		Bivariate Regression Analysis		Multivariate Regression Analysis	
	Negative	Positive	P-value	COR (95% CI)	P-value	AOR (95%) CI)
Age of participant						
Birth -1 month	208(90.8%)	21(9.17%)	**0.016**	2.928(1.218–7.038)	0.093	3.277(0.819–13.108)
>1mth-<13 years	562(95.7%)	25(4.3%)	0.559	1.29(0.55–3.028)	0.057	2.47(0.972–6.279)
13-<45 years	443(96.3%)	17(3.7%)	0.815	1.113(0.454–2.726)	0.709	1.197(0.466–3.072)
>45 years	203(96.7%)	7(3.33%)	0.009		0.930	
Patient admission ward						
B6	176(90.3%)	19(9.74%)	**0.004**	3.904(1.526–9.986)		
C/W	344(97.5%)	9(2.55%)	0.918	0.946(0.332–2.696)		
B4	107(89.9%)	12(10.1%)	**0.006**	4.056(1.482–11.103)		
D7	139(96.5%)	5(3.47%)	0.669	1.301(0.39–4.344)		
EOPD	219(98.6%)	3(1.35%)	0.325	0.495(0.122–2.006)		
B7	36(87.8%)	5(12.2%)	**0.011**	5.023(1.456–17.325)		
C7	58(93.5%)	4(6.45%)	0.168	2.494(0.681–9.133)		
D8	71(95.9%)	3(4.05%)	0.556	1.528(0.373–6.269)		
B5	49(92.5%)	4(7.55%)	0.103	2.952(0.803–10.861)		
Other	217(97.3%)	6(2.69%)	0.000			
Patient admission department						
Medical	710(97.4%)	19(2.6%)	0.000		0.019	
Emergency	70(98.6%)	1(1.41%)	0.544	0.534(0.07–4.048)	0.952	1.066(0.134–8.51)
MICU	68(93.2%)	5(6.85%)	**0.051**	2.748(0.995–7.59)	0.963	0.973(0.308–3.073)
NICU	161(90.4%)	17(9.55%)	**<0.001**	3.946(2.006–7.76)	0.462	1.549(0.483–4.974)
General surgery	38(82.6%)	8(17.39%)	**<0.001**	7.867(3.237–19.122)	**0.001**	5.102(1.938–13.435)
SICU	27(79.4%)	7(20.59%)	**<0.001**	9.688(3.754–25)	**0.007**	4.787(1.533–14.951)
Hematology	293(96.1%)	12(3.93%)	0.257	1.53(0.734–3.193)	0.576	1.25(0.571–2.738)
Others	49(98%)	1(2%)	0.794	0.763(0.1–5.816)	0.627	0.599(0.076–4.72)
Patient length of admission						
1–2 days	569(98.4%)	9(1.56%)	0.000			
3–4 days	201(94.4%)	12(5.63%)	**0.003**	3.774(1.567–9.092)		
>5days	646(92.9%)	49(7.05%)	**<0.001**	4.795(2.335–9.849)		
ICU admission						
No	1150(97%)	35(2.95%)	0.000			
Yes	266(88.4%)	35(11.63%)	**0.000**	4.323(2.656–7.037)	**0.008**	2.611(1.288–5.292)
Antibiotic treatment before ten days of sample collection						
No	667(97.5%)	17(2.49%)	0.000			
Yes	749(93.4%)	53(6.61%)	**0.000**	2.776(1.592–4.842)	**0.023**	2.045(1.104–3.787)
Instrument of inserted device usage during medical care						
No	613(99%)	6(0.97%)	0.000			
Yes	803(92.6%)	64(7.38%)	**<0.001**	8.143(3.503–18.926)	**0.001**	4.297(1.773–10.412)
Using a suction machine during medical care						
No	696(93.3%)	50(6.7%)	0.000			
Yes	107(88.4%)	14(11.57%)	**0.071**	2.285(0.932–5.602)	**0.040**	1.974(1.032–3.772)
The insertion of an intravenous line during medical care						
No	82(96.5%)	3(3.53%)	0.000			
Yes	721(92.2%)	61(7.8%)	**0.036**	3.812(1.091–13.321)	**0.045**	3.502(1.027–11.944)
The insertion of a drainage tube during medical care						
No	778(93%)	59(7.05%)	0.000			
Yes	25(83.3%)	5(16.67%)	**0.030**	3.296(1.122–9.678)	**0.032**	3.22(1.108–9.36)
Patients having chronic wound infection on the skin during medical care						
No	907(96.4%)	34(3.61%)	0.000			
Yes	18(81.8%)	4(18.18%)	0.102	2.949(0.807–10.775)	**0.030**	3.826(1.136–12.883)
The patient has a chronic urinary tract infection during medical care						
No	892(96.5%)	32(3.46%)	0.000			
Yes	33(84.6%)	6(15.38%)	**0.017**	3.678(1.261–10.73)	**0.007**	3.887(1.437–10.516)

Wards and their department = B6, NICU; C/W, casualty; B4, SICU, MICU/PMICU; D7, Pediatrics Hematology; EOPD Emergency outpatient department; B7, pediatrics medical & surgery; C7, Pediatrics Medical; D8, Medical; B5, Medical & Neurosurgery; MBL: Metallo beta-lactamase; Values in bold are statistically significant.

Mode of delivery of neonates was associated with carbapenemase and MBL producer bacterial infection (p = 0.036), caesarean section delivery was almost 10 times more likely to result in acquiring these bacteria than normal delivery (Table 4).

**Table 4:** Bivariate and multivariate logistic regression analysis of neonatal demographic and other parameters, taken as predictive variables for carbapenemase and MBL-producing bacterial infection among BSI patients.

Variables for neonates only	Carbapenemase and MBL		Bivariate Regression Analysis		Multivariate Regression Analysis	
	Negative	Positive	P-value	COR (95%CI)	P-value	AOR (95%CI)
The educational level of family						
Illiterate	30(96.8%)	1(3.23%)	0.009		0.025	
Read and write	65(97%)	2(2.99%)	**0.042**	0.092(0.009–0.912)	0.076	0.115(0.011–1.25)
Primary school	54(94.7%)	3(5.26%)	**0.008**	0.085(0.014–0.519)	**0.012**	0.088(0.013–0.589)
Secondary school	49(81.7%)	11(18.33%)	**0.024**	0.153(0.03–0.781)	**0.049**	0.176(0.031–0.993)
Higher education	11(73.3%)	4(26.67%)	0.473	0.617(0.165–2.306)	0.524	0.628(0.15–2.625)
Have a history of neonatal incubation						
No	57(91.9%)	5(8.06%)	0.000			
Yes	152(90.5%)	16(9.52%)	0.733	1.2(0.42–3.427)		
Neonatal mode of delivery					
NVD	156(94%)	10(6.02%)	0.000			
C/S	53(82.8%)	11(17.19%)	**0.012**	3.238(1.302–8.054)		
Gestational age during delivery					
>37 weeks	127(91.4%)	12(8.63%)	0.000			
<37 weeks	82(90.1%)	9(9.89%)	0.746	1.162(0.469–2.879)		
Premature rupture of the membrane during delivery						
>18 hours	44(97.8%)	1(2.22%)	0.006		0.027	
<18 hours	146(91.8%)	13(8.18%)	0.194	3.918(0.498–30.79)	0.327	2.87(0.348–23.68)
No rupture (C/S)	19(73.1%)	7(26.92%)	**0.012**	16.21(1.86–141.02)	**0.036**	10.94(1.17–102.6)
Body weight range during delivery						
Normal	114(94.2%)	7(5.79%)	0.179		0.186	
Low	93(86.9%)	14(13.08%)	0.064	2.452(0.95–6.325)	0.067	2.61(0.94–7.27)
High	2(100%)	0(0%)	0.999		0.999	
History of maternal fever or infection during pregnancy						
No	189(90.4%)	20(9.57%)	0.000			
Yes	20(95.2%)	1(4.76%)	0.476	0.473(0.06–3.709)		

MBL: Metallo beta-lactamase; NVD: normal vaginal delivery; C/S: caesarean section; Values in bold are statistically significant.

### Factor associated with ESBL and AmpC-BL producer gram-negative bacterial infection

The multivariate regression analysis of the study demonstrated significant associations between ESBL and AmpC-BL-producing GNB infection and insertion of instruments into the body during medical care (p = <0.001) and chronic illnesses (p = 0.027). Patients having UTI were almost 4 times more likely to acquire ESBL and AmpC BLs producer bacteria than those who don’t have UTI (p = 0.001). Again, patients having respiratory tract infection (RTI) (p = 0.001) and tissue sarcoma (p = 0.018) have a double chance to acquire ESBL and AmpC-BL-producing bacterial infection than those who don’t have them. There was a significant association between the age group and ESBL and AmpC-BL-producing GNB infection (p< 0.0001). Age less than one month was 3.5 times more likely to acquire ESBL and AmpC-BL-producing GNB infection than > 45 years of age (p = <0.001). There was a significant association between ESBL and AmpC-BL-producing GNB infection and patients’ admission departments (p< 0.0001). Among patients admitted in wards of B6 (NICU) (p = <0.001), D7 (Pediatrics Hematology) (p = 0.044), and B7 (pediatrics, medical & surgery) (p = 0.014) were almost 15, 2.5 and 4.5 times more likely to acquire ESBL and AmpC producer bacteria than those admitted in other wards., respectively (Table 5).

**Table 5:** Bivariate and multivariate logistic regression analysis of demographic and other parameters, taken as predictive variables of infection with ESBL and AmpC-BL producing GNB among BSI patients.

Variables	ESBL & AmpC-BL		Bivariate Regression Analysis		Multivariate Regression Analysis	
	Negative	Positive	P-value	COR (95% CI)	P-value	AOR (95% CI)
Age of participant						
Birth -1 month	181(79.04%)	48(20.96%)	**<0.001**	3.448(1.866–6.371)		
>1mth-<13 years	546(93.02%)	41(6.98%)	0.939	0.976(0.528–1.803)		
13-<45 years	445(96.74%)	15(3.26%)	0.028	0.438(0.21–0.914)		
>45 years	195(92.86%)	15(7.14%)	0.000			
Patient admission ward						
B6	149(76.41%)	46(23.59%)	**<0.001**	8.297(3.81–18.087)	**<0.001**	15.15(5.61–40.922)
C/W	336(95.18%)	17(4.82%)	0.482	1.36(0.577–3.206)	0.182	1.81(0.757–4.328)
B4	110(92.44%)	9(7.56%)	0.115	2.199(0.826–5.857)	0.17	2.005(0.743–5.413)
D7	130(90.28%)	14(9.72%)	**0.020**	2.894(1.182–7.087)	**0.044**	2.524(1.025–6.211)
EOPD	210(94.59%)	12(5.41%)	0.358	1.536(0.615–3.833)	0.193	1.848(0.733–4.655)
B7	36(87.8%)	5(12.2%)	**0.028**	3.733(1.156–12.05)	**0.014**	4.565(1.36–15.286)
C7	57(91.94%)	5(8.06%)	0.146	2.357(0.743–7.482)	0.121	2.515(0.784–8.068)
D8	71(95.95%)	3(4.05%)	0.854	1.136(0.293–4.397)	0.934	1.059(0.272–4.117)
B5	53(100%)	0(0%)	0.997	0(0–0)	0.997	0(0–0)
Other	215(96.41%)	8(3.59%)	0.000			
Patient admission department						
Medical	686(94.1%)	43(5.9%)	0.000			
Emergency	68(95.77%)	3(4.23%)	0.565	0.704(0.213–2.329)		
MICU	68(93.15%)	5(6.85%)	0.744	1.173(0.45–3.061)		
NICU	138(77.53%)	40(22.47%)	**<0.001**	4.624(2.897–7.382)		
General surgery	43(93.48%)	3(6.52%)	0.862	1.113(0.332–3.734)		
SICU	31(91.18%)	3(8.82%)	0.487	1.544(0.454–5.253)		
Hematology	287(94.1%)	18(5.9%)	0.998	1.001(0.567–1.764)		
Others	46(92%)	4(8%)	0.548	1.387(0.477–4.033)		
ICU admission						
No	1114(94.01%)	71(5.99%)	0.000			
Yes	253(84.05%)	48(15.95%)	**<0.001**	2.977(2.014–4.4)		
Antibiotic treatment before in ten days						
No	1268(93.17%)	93(6.83%)	0.000			
Yes	99(79.2%)	26(20.8%)	**<0.001**	3.581(2.215–5.79)		
Instrument of inserted device usage during medical care						
No	593(95.8%)	26(4.2%)	0.000			
Yes	774(89.27%)	93(10.73%)	**<0.001**	2.74(1.751–4.288)	**<0.001**	2.369(1.476–3.802)
The patient has a chronic illness						
No	901(93.56%)	62(6.44%)	0.000			
Yes	466(89.1%)	57(10.9%)	**0.003**	1.778 (1.220–2.591)	**0.027**	2.128(1.088–4.162)
The patient has a chronic urinary tract infection						
No	870(94.16%)	54(5.84%)	0.000			
Yes	31(79.49%)	8(20.51%)	**<0.001**	6.324(2.46–16.252)	**0.001**	4.102(1.778–9.466)
The patient has a chronic respiratory tract infection						
No	822(94.16%)	51(5.84%)	0.000			
Yes	79(87.78%)	11(12.22%)	**0.004**	3.322(1.465–7.533)	**0.018**	2.35(1.156–4.777)
The patient has chronic tissue Sarcomas						
No	791(94.17%)	49(5.83%)	0.000			
Yes	110(89.43%)	13(10.57%)	**0.037**	2.778(1.062–7.268)	**0.022**	2.153(1.116–4.154)

Wards and their department = B6, NICU; C/W, casualty; B4, SICU, MICU/PMICU; D7, Pediatrics Hematology; EOPD Emergency outpatient department; B7, pediatrics medical & surgery; C7, Pediatrics Medical; D8, Medical; B5, Medical & Neurosurgery; ESBL: Extended-spectrum beta-lactamase; AmpC-BL: AmpC β-lactamase; Values in bold are statistically significant.

Neonatal incubation has an association (p = 0.006) with ESBL and AmpC-BL-producing bacterial infection (Table 6).

**Table 6:** Bivariate and multivariate logistic regression of neonatal demographic and other parameters, taken as predictive variables for BSI by GNB that produce ESBL and AmpC-BL.

Variables for neonates only	ESBL + AmpC-BL		Bivariate Regression Analysis		Multivariate Regression Analysis	
	Negative	Positive	P-value	COR (95%CI)	P-value	AOR (95%CI)
The educational level of family						
Illiterate	23(74.19%)	8(25.81%)	0.073			
Read and write	61(91.04%)	6(8.96%)	0.666	1.391(0.311–6.231)		
Primary School	44(77.19%)	13(22.81%)	0.228	0.393(0.086–1.795)		
Secondary school	42(70%)	18(30%)	0.816	1.182(0.289–4.833)		
Higher Education	12(80%)	3(20%)	0.444	1.714(0.431–6.817)		
Have a history of neonatal incubation						
No	57(91.94%)	5(8.06%)	0.000			
Yes	125(74.4%)	43(25.6%)	**0.006**	3.92(1.475–10.42)	**0.006**	4.0(1.5–10.69)
Neonatal mode of delivery						
NVD	134(80.72%)	32(19.28%)	0.000			
C/S	48(75%)	16(25%)	0.340	1.396(0.704–2.768)		
Gestational age during delivery						
>37 weeks	113(81.29%)	26(18.71%)	0.000			
<37 weeks	69(75.82%)	22(24.18%)	0.319	1.386(0.729–2.633)		
Premature rupture of the membrane during delivery						
>18 hours	38(84.44%)	7(15.56%)	0.508			
<18 hours	125(78.62%)	34(21.38%)	0.391	1.477(0.606–3.599)		
No rupture	19(73.08%)	7(26.92%)	0.251	2.0(0.612–6.532)		
History of maternal fever or infection during pregnancy						
No	168(80.38%)	41(19.62%)	0.000			
Yes	14(66.67%)	7(33.33%)	0.147	2.049(0.777–5.401)	0.129	2.17(0.80–5.9)

ESBL: Extended-spectrum beta-lactamase; AmpC-BL: AmpC β-lactamase; NVD: normal vaginal delivery; C/S: caesarean section; Values in bold are statistically significant.

### Factors associated with MDR gram-negative bacterial infection

A total of 215(51.56%) patients who acquired MDR GNB were analyzed for the associated factors with the infection acquired. The multivariate regression analysis of the study revealed that patient admission department (MICU(p = 0.027), NICU (p = 0.023), General Surgery (p = 0.001)), instrument usage during medical care (p = <0.001), (an intravenous line (p = 0.029), and drainage tube (p = 0.033)) and having a chronic illness (p = 0.041) like RTI (p = 0.027) have significant proportion of MDR GNB bacteria. Neonates with a history of neonatal incubation were identified to have the possibility of acquiring MDR bacteria three times more than neonatal patients who are not treated in neonatal incubation. Chronically ill patients, those with UTIs were five times more likely to acquire MDR bacteria than those without UTIs. Neonates delivered by Caesarean section were more likely to acquire MDR bacteria than those delivered with normal vaginal delivery (S2 Table).

## Discussion

Comprehending the so-called "nightmare" of MDR bacteria is key to the successful treatment and control of hospital-acquired infection [38]. Therefore, this study was intended to identify the magnitude of MDR and their phenotypic drug-resistance mechanisms including the production of ESBL, AmpC-BL, MBL, and Carbapenemase along with their associated factors among patients with GNB bloodstream infection in a tertiary care hospital in Ethiopia. A high percentage of ESBL + AmpC-BL bacteria (67.1%) were identified meanwhile carbapenemase + MBL was found to be 30.7%. In this study, the rate of BSI was 28.1% which is comparable to other related studies conducted in Ethiopia with prevalence rates of 28.0% [39], 27.9% [40], and 25.8% [41]. However, the current result was higher than rates reported elsewhere18.2% [42], 15.8% [43], and lower than other studies conducted in Ethiopia which were 32.8% [19], and 39.2% [44]. A lower percentage was also reported from Bangladesh 13.6% [45]. Several confounding factors including differences in infection prevention practices, crowdedness of the hospitals, study settings, study’s design, study area, patient type, clinical situation, etiological factors, and seasonal variations could account for the differences observed.

The higher prevalence of GNB (54.2%) was also reported in the present study, which accords with earlier Ethiopian studies [44] 52.3%, [40] 51.8%; however, it was higher than other reports from the country such as 31% [42], and 22.6% [19]. The GNBs are becoming the major etiology of BSI in hospitalized patients [44, 46, 47]. In this research, Klebsiella pneumoniae, Acinetobacter spp., and Escherichia coli were the three most common pathogens isolated. This was similar to a study from Paris and CDC reports that also identified these three pathogens as the most common isolates [48, 49]. This could be due to the sharing of drug-resistant genetic materials associated with ESBL, AmpC-BL, and Carbapenemase-producing genes among these bacteria [4, 44, 46, 47].

The total percentage (84.4%) of bacteria in the current study that produced carbapenemase, MBL, ESBL, and AmpC-BL was higher than the study done in Addis Ababa Ethiopia (70%) [13]. However, this was lower than the study conducted in Nigeria (98.8%) [50]. Of 195 drug resistance enzyme-producing bacteria, we found 15.9% that produced two or more enzymes which were lower than the Nigerian report showing co-production of ESBL, AmpC-BL, and/or Carbapenemase in 69.6% of isolates [50]. Whereas when it comes to the rate of single enzyme production our study showed ESBL (52.8%), carbapenemase (25.1%), AmpC-BL (14.3%), and MBL (5.6%) producers which was comparable with the study from Nigeria with rates for ESBL (53.9%) and AmpC-BL (20.9%). The carbapenemase and MBL-producing bacteria in this study were 30.7%, which was higher than studies conducted in Nigeria that was 25.2% [50], and Ethiopia which was 2% [13] and 12.12% [15]. These indicate that the prevalence of GNB-producing drug-hydrolyzing enzymes is increasing over time. Carbapenemases, with versatile hydrolytic capacity against β-lactams, are now an important mechanism of drug resistance in GNB [51]. In this study, 30.7% of carbapenemase-producing GNB was higher than that of a study done in Latin America with 20.8% [52]. This could be due to the high percentage of *Acinetobacter baumannii* which accounts for 76.5% of the total possible PDR and 63.8% of *Acinetobacter spp* isolated in this study were carbapenem drug-resistant. The genes encoding for the acquired carbapenemase production were associated with a high potential for dissemination [51]. Carbapenem-resistant *Acinetobacter spp*. that carry mobile genetic elements were associated with nosocomial infection in our study settings and some of these bacteria were pan-drug-resistant [34]. Therefore, special attention must be given in our hospital to all types of *Klebsiella pneumoniae* that were associated with carbapenem resistance and possible PDR *Acinetobacter spp* identified, more specifically about their transmission dynamics.

The prevalence of ESBL and AmpC-BL-producing bacteria was 67.1%, comparable to a study conducted in Addis Ababa, which had reported 67% [13] and 78.6% [15]. In our study, the percentage (52.8%) ESBL producing GNB isolated from patients with BSI was similar to the nine-year retrospective study conducted in Ghana which accounts for 50.2% [53]. A lower result was also reported by previous similar studies conducted in Ethiopia (38.6%) [12], Asia (47%) [54], and Thailand (37.8%) [55]. The difference could account for differences in the research methodology, sample type, geographical variation, and hospital infection prevention practice.

In our study, *Klebsiella pneumoniae* was the highest drug-resistance enzyme-producing bacteria accounting for 36.7% followed by *Acinetobacter* (21.7%). The *Acinetobacter spp*. was the high producer of carbapenemase and MBL producers with 53.2% and 10.6% prevalence, respectively. According to a study conducted on urine samples in Nigeria, the percentage of *Klebsiella pneumoniae* strains resistant to cephalosporins due to ESBL production increased from 18.2% in 2010 to 29.4% in 2011 and further to 34.1% in 2012 [56]. A study conducted in Kenya revealed *Klebsiella pneumoniae*, *E*. *coli*, and *Acinetobacter baumannii* had resistance to cephalosporins with a range of 57–84%, 16–43%, and 81%, respectively [57]. The systematic review of 20 years conducted in China on BSI hospitalized non-ICU patients with BSI had shown carbapenem-resistant *Klebsiella pneumoniae* to be below 5% in the years 1998–2012 but this figure had increased to 34.9% during 2013–2017. In contrast, sadly, the detection rate for carbapenem-resistant *Klebsiella pneumoniae* in ICU patients has increased from 0% in 2013 to 75% in 2016 [58].

Having a double drug resistance enzyme-producing bacteria implies having a high drug resistance capacity for the bacteria and this in turn worsens the success rate of the treatment to the worst level. In this study, about 15.9% of the isolated bacteria produced more than one drug hydrolyzing enzyme that was higher than that of China, which reported 7% of ESBL-producing *Klebsiella pneumoniae* isolates manifested “cross-resistance to carbapenems [56]. In this research, *Klebsiella pneumoniae*, *Acinetobacter spp*., and *Klebsiella oxytoca* were the three dominant bacteria that produced more than one drug hydrolyzing enzymes. This implies that *Klebsiella pneumoniae*, *Klebsiella oxytoca*, *Acinetobacter spp*, and *Escherichia coli* were becoming aggressive drug resistance bacteria. In addition to this, having more than one drug-hydrolyzing enzymes strengthens the bacterial pathogens to be more resistant to antibiotics, increased treatment failure rate, longer hospital stays, high economic burden, and unpredicted emotional costs to patients.

The higher percentage of Carbapenemase and MBL producer GNB reported in general surgery and SICU department, were almost five times more likely to be contagious than other admission departments. Neonates delivered by caesarean section were almost 10 times more likely to acquire carbapenemase and MBL producer bacteria than those delivered by normal procedure. In recent years, the rate of caesarean section happening is increasing, and whether that is indicated or not is an issue but the fact that the procedure is a risk factor that exposes the neonates to MDR bacteria calls for serious attention in the national maternal health programs. Patients that pass through surgical procedures including caesarean section had an association with carbapenem infections. These results will be directly associated with a lack of infection prevention protocol implementation in the caesarean section room and sterilization of medical instruments used during surgical procedures. Fumigation of the room, proper sterilization technique for the instruments, and standard surgical procedure should be implemented. These factors were also found in another study conducted in Latin America where ICU admission and general surgery have an association with carbapenem drug-resistant bacterial infection [52].

The other major factors associated with carbapenem resistance bloodstream infection in the current study were the usage of instruments especially inserted devices during medical care were almost four times more likely to be infectious for carbapenemase and MBL producer bacteria. Usage of antibiotic treatment ten days before sample collection was also associated with carbapenemase and MBL producer bacteria. The misuse of antibiotics will be one of the causes of this problem. From patients with chronic illness, patients with wound infection on the skin and UTI were almost 4 times more likely to acquire carbapenemase and MBL producer bacteria than those that do not have a wound and UTI. Wound infection on the skin was one of exposing factors for nosocomial infection due to the lack of mechanical barrier of the skin. The association between wound infection and UTI with Carbapenemase producer GNB BSI was also reported in another study [52]. The instruments used for medical care like a suction machine, an intravenous line, and a drainage tube were associated with Carbapenemase and MBL-producing bacterial infection. Therefore, surgical materials and indwelling medical equipment during medical care should be monitored for proper sterilization.

The NICU wards were almost 15 times more likely to acquire ESBL and AmpC-BL producer GNB in this study. The room temperature which is favorable for microbial growth, high workload, and crowdedness could be a major problem in NICU BSI. In addition, plasmid transfer genes that play a great role in nosocomial infection are common in GNB, especially in *E*. *coli* and *Klebsiella species* found in these wards. As per the research conducted in 11 African countries among neonates, GNB was the predominant cause of early-onset neonatal sepsis, with a high prevalence of ESBL-producing bacteria [59]. This implies that NICU was more affected by third-generation cephalosporin-resistant bacteria meanwhile, the caesarean section delivery room was more affected by Carbapenem-resistant bacteria.

Among chronic illness patients those having UTI were almost 4 times more likely to acquire ESBL and AmpC-BL producer bacteria than those who don’t have UTI. The patients with respiratory tract infection(RTI) and tissue sarcomas were also two times more likely to acquire ESBL and AmpC-BL producers than the others. Immune suppression and prolonged admission in chronic illness patients could be the exposing factors for hospital-acquired infection.

The prevalence of MDR, XDR, and PDR was indicated in our previous article [37]. The ICU department, neonate’s instrument usage during medical care, chronic illness, UTI, RTI, and history of incubation was the predominant factor for having the highest MDR GNB infection. Another study conducted in Australia showed a three-fold increased risk of death in the ICU department [60]. Likewise, another study in the USA, Australia, and India showed that MDR-GNBs were typically isolated in ICUs [61–63]. Our result is also supported by the result of the study conducted in Vellore, South India, Ghana, and Australia where neonatal ICU admission has an association with MDR infection [53, 60, 62]. The study done in Greece showed that PDR in GNB was typically isolated in ICUs [64]. Surgery and surgical admission were identified as independent risk factors in America and Australia for MDR-BSI [60, 65]. A two-fold exposure to MDR infection was also found in patients with a medical instrument used during medical care [62]. A study conducted in Thailand showed RTI and wound infection as significant risk factors for MDR-GNB infection [55]. Another research conducted in the USA also indicated the usage of urinary catheters as a risk factor for the development of MDR in GNB [66]. The neonatal incubation and mode of delivery by caesarean section had three and two-fold exposure to MDR BSI [51, 67]. From this result, we can recommend sterilization of the medical equipment used for the ICU and surgical procedures, and the infection prevention protocol should be strengthened.

## Conclusion

In this study, a large number of GNBs were drug-resistant enzyme producers. Two or more drug-resistant enzymes were produced by 15.9% GNB. High carbapenemase and MBL producer bacteria were found in this study. Most patients in neonatal ICUs were susceptible to ESBL and AmpC-BL-producing GNB, whereas, in general surgery, caesarean section delivery room, and SICU patients were more susceptible to carbapenemase-producing GNB. The suction machines, intravenous lines, and drainage tubes were the major cause of acquiring carbapenemase and MBL producer bacteria. Among chronic illnesses, wound infection, and UTI were associated with carbapenemase and MBL producer bacteria. The ESBL and AmpC-BL producers were also associated with UTI, RTI, and tissue sarcomas. The 7.4% of possible PDR bacteria was an alarming result in this study. *Klebsiella pneumoniae* and *Acinetobacter spp*. were the most prevalent bacteria with XDR and possible PDR, respectively. The hospital management would extend their effort to address the concern of infection prevention protocols in the NICU department in particular and the hospital in general. Special attention should be given to all types of *Klebsiella penumoniae* and possible PDR *Acinetobacter spp* transmission dynamics, drug resistance genes, and virulence factors.

## Limitation

The obligate anaerobes were not identified in this study. We used conventional biochemical tests rather than API 20 or MALDI-TOF mass spectrometry because of unavailability and high expense.

## Supporting information

S1 Table Gram-negative bacteria and their enzyme-producing drug resistance mechanism from BSI suspected patients. (DOCX)

S2 Table Factors associated with MDR prevalence crude and adjusted odds ratio of BSI suspected patients. Bivariate and multivariate logistic regression of demographic and other parameters, taken as predictive variables for BSI patients with MDR acquired 215 patients compared with 1271 BSI patients.
(DOCX)
