# Supplementary material for: High burden of ESBL and carbapenemase-producing gram-negative bacteria in bloodstream infection patients at a tertiary care hospital in Addis Ababa, Ethiopia
Source: PLoS One. 2023 Jun 27;18(6):e0287453. doi: 10.1371/journal.pone.0287453 (PMC10298750; doi:10.1371/journal.pone.0287453)
Supplement: S1 Table — (DOCX) [file pone.0287453.s001.docx]

**Supporting information**

S1 Table. Gram-negative bacteria and their enzyme-producing drug resistance mechanisms from BSI suspected patients.

| Bacteria identified | Negative | ESBL | AmpC-BL | CP | MBL | T. DRM | GNB | |
| --- | --- | --- | --- | --- | --- | --- | --- | --- |
|  | **N(%)** | **N(%)** | **N(%)** | **N(%)** | **N(%)** | **N(%)** | **Total** |  |
| *K. pneumoniae* | 3(4.2%) | 43(60.6%) | 6(8.5%) | 15(21.1%) | 4(5.6%) | 68(95.8%) | 71 |  |
| *Acinetobacter spp* | 6(13.0%) | 2(4.3%) | 9(19.6%) | 24(52.2%) | 5(10.9%) | 40(87.0%) | 46 |  |
| *Escherichia coli* | 9(25.0%) | 13(36.1%) | 8(22.2%) | 4(11.1%) | 2(5.6%) | 27(75.0%) | 36 |  |
| *Klebsiella oxytoca* | 2(7.4%) | 16(59.3%) | 4(14.8%) | 5(18.5%) | 0(0.0%) | 25(92.6%) | 27 |  |
| *Escherichia coli(A-D)* | 2(18.2%) | 6(54.5%) | 1(9.1%) | 1(9.1%) | 1(9.1%) | 9(81.8%) | 11 |  |
| *Citrobactor diversus* | 3(60.0%) | 2(40.0%) | 0(0.0%) | 0(0.0%) | 0(0.0%) | 2(40.0%) | 5 |  |
| *Pseudomonas spp* | 1(20.0%) | 0(0.0%) | 2(40.0%) | 2(40.0%) | 0(0.0%) | 4(80.0%) | 5 |  |
| *K. rhinoscleroma* | 2(50.0%) | 0(0.0%) | 0(0.0%) | 1(25.0%) | 1(25.0%) | 2(50.0%) | 4 |  |
| *Serratia marcescens* | 0(0.0%) | 1(25.0%) | 1(25.0%) | 2(50.0%) | 0(0.0%) | 4(100.0%) | 4 |  |
| *Klebsiella ozaenae* | 2(66.7%) | 0(0.0%) | 1(33.3%) | 0(0.0%) | 0(0.0%) | 1(33.3%) | 3 |  |
| *Enterobacter cloacae* | 1(50.0%) | 0(0.0%) | 0(0.0%) | 1(50.0%) | 0(0.0%) | 1(50.0%) | 2 |  |
| *E. agglomerans* | 1(50.0%) | 1(50.0%) | 0(0.0%) | 0(0.0%) | 0(0.0%) | 1(50.0%) | 2 |  |
| *Morganella morganii* | 1(50.0%) | 0(0.0%) | 0(0.0%) | 1(50.0%) | 0(0.0%) | 1(50.0%) | 2 |  |
| *Proteus mirabilis* | 1(50.0%) | 0(0.0%) | 0(0.0%) | 1(50.0%) | 0(0.0%) | 1(50.0%) | 2 |  |
| *Providencia rettgeri* | 0(0.0%) | 2(100.0%) | 0(0.0%) | 0(0.0%) | 0(0.0%) | 2(100.0%) | 2 |  |
| *E. aerogenes* | 0(0.0%) | 0(0.0%) | 1(100.0%) | 0(0.0%) | 0(0.0%) | 1(100.0%) | 1 |  |
| *Salmonella typhi* | 1(100.0%) | 0(0.0%) | 0(0.0%) | 0(0.0%) | 0(0.0%) | 0(0.0%) | 1 |  |
| Total | 35(15.6%) | 86(38.4%) | 33(14.7%) | 57(25.4%) | 13(5.8%) | 189(84.4%) | 224 |  |

DRM: Drug resistance mechanisms; ESBL: Extended-spectrum beta-lactamase; AmpC-BL: AmpC beta-lactamase; CP: Carbapenemase; MBL: Metallo-beta-lactamases; GNB: Gram-negative bacteria;
