# Supplementary material for: High burden of ESBL and carbapenemase-producing gram-negative bacteria in bloodstream infection patients at a tertiary care hospital in Addis Ababa, Ethiopia
Source: PLoS One. 2023 Jun 27;18(6):e0287453. doi: 10.1371/journal.pone.0287453 (PMC10298750; doi:10.1371/journal.pone.0287453)
Supplement: S2 Table — Bivariate and multivariate logistic regression of demographic and other parameters, taken as predictive variables for BSI patients with MDR acquired 215 patients compared with 1271 BSI patients. (DOCX) [file pone.0287453.s002.docx]

S2 Table. Factors associated with MDR prevalence crude and adjusted odds ratio of BSI suspected patients. Bivariate and multivariate logistic regression of demographic and other parameters, taken as predictive variables for BSI patients with MDR acquired 215 patients compared with 1271 BSI patients.

| **Variables** | **Bacterial MDR prevalence** | | **Bivariate logistic regression analysis** | | | **Multivariate logistic regression analysis** | |
| --- | --- | --- | --- | --- | --- | --- | --- |
|  | **MDR N(**%) | **No MDR N(%)** | **P-value** | **COR (95% CI)** | | **P-value** | **AOR (95% CI)** |
| Age of respondent | |  |  |  | |  |  |
| Birth to 1 month | 74(32.3%) | 155(67.7%) | **<0.001** | 3.379(2.059-5.544) | | **0.007** | 3.149(1.376-7.209) |
| >1mon-<13 years | 78(13.3%) | 509(86.7%) | 0.738 | 1.084(0.675-1.743) | | 0.172 | 1.426(0.857-2.371) |
| 13-<45 years | 37(8%) | 423(92%) | 0.076 | 0.619(0.364-1.052) | | 0.125 | 0.649(0.373-1.128) |
| >45 years | 26(12.4%) | 184(87.6%) | 0.000 |  | | 0.000 |  |
| Admission admission ward | |  |  |  | |  |  |
| B6 | 72(36.9%) | 123(63.1%) | **<0.001** | 7.573(4.215-13.61) | |  | |
| C/W | 32(9.1%) | 321(90.9%) | 0.425 | 1.290(0.69-2.410) | |  |  |
| B4 | 22(18.5%) | 97(81.5%) | **0.002** | 2.934(1.475-5.836) | |  |  |
| D7 | 22(15.3%) | 122(84.7%) | **0.015** | 2.333(1.18-4.613) | |  |  |
| EOPD | 18(8.1%) | 204(91.9%) | 0.711 | 1.142(0.567-2.30) | |  |  |
| B7 | 10(24.4%) | 31(75.6%) | **0.001** | 4.173(1.739-10.02) | |  |  |
| C7 | 10(16.1%) | 52(83.9%) | **0.035** | 2.488(1.067-5.801) | |  |  |
| D8 | 8(10.8%) | 66(89.2%) | 0.323 | 1.568(0.642-3.829) | |  |  |
| B5 | 5(9.4%) | 48(90.6%) | 0.578 | 1.348(0.471-3.859) | |  |  |
| Other | 16(7.2%) | 207(92.8%) | 0.000 |  | |  |  |
| Patient admission department | | |  |  |  | |  |
| Medical | 71(9.7%) | 658(90.3%) | 0.000 |  | | 0.017 |  |
| Emergency | 6(8.5%) | 65(91.5%) | 0.725 | 0.855(0.358-2.045) | | 0.713 | 1.188(0.474-2.975) |
| MICU | 11(15.1%) | 62(84.9%) | 0.156 | 1.644(0.828-3.266) | | 0.637 | 1.188(0.581-2.429) |
| NICU | 63(35.4%) | 115(64.6%) | **<0.001** | 5.077(3.428-7.52) | | **0.027** | 2.205(1.093-4.451) |
| G. Surgery | 11(23.9%) | 35(76.1%) | **0.004** | 2.913(1.417-5.986) | | **0.023** | 2.479(1.133-5.422) |
| SICU | 10(29.4%) | 24(70.6%) | **0.001** | 3.862(1.775-8.401) | | **0.001** | 4.013(1.722-9.35) |
| Hematology | 38(12.5%) | 267(87.5%) | 0.195 | 1.319(0.868-2.005) | | 0.677 | 1.104(0.694-1.755) |
| Others | 5(10%) | 45(90%) | 0.952 | 1.03(0.396-2.678) | | 0.809 | 0.885(0.328-2.385) |
| Length of admission | |  |  |  | |  |  |
| 1-2 days | 70(12.1%) | 508(87.9%) | 0.036 |  | | 0.498 |  |
| 3-4 days | 27(12.7%) | 186(87.3%) | 0.830 | 1.053(0.655-1.694) | | 0.311 | 0.763(0.452-1.288) |
| >5days | 118(17%) | 577(83%) | **0.015** | 1.484(1.079-2.041) | | 0.941 | 1.016(0.676-1.526) |
| ICU admission |  |  |  |  | |  |  |
| No | 126(10.6%) | 1059(89.4%) | 0.000 |  | |  |  |
| Yes | 89(29.6%) | 212(70.4%) | **<0.001** | 3.528(2.591-4.805) | |  | |
| Body temperature | | |  |  |  | |  |
| <36.5 | 36(21.2%) | 134(78.8%) | 0.000 |  | | 0.655 |  |
| 36.6-37.5 | 14(17.7%) | 65(82.3%) | 0.527 | 0.802(0.404-1.59) | | 0.381 | 0.701(0.316-1.554) |
| >37.6 | 165(13.3%) | 1072(86.7%) | **0.007** | 0.573(0.383-0.857) | | 0.477 | 0.731(0.308-1.734) |
| Antibiotic treatment 10 days before? | | |  |  |  | |  |
| No | 83(12.1%) | 601(87.9%) | 0.000 |  | |  |  |
| Yes | 132(16.5%) | 670(83.5%) | **0.019** | 1.427(1.061-1.918) | | 0.15 | 1.315(0.906-1.908) |
| Instrument usage during med care? | | |  |  |  | |  |
| No | 42(6.8%) | 577(93.2%) | 0.000 |  | |  |  |
| Yes | 173(20%) | 694(80%) | **<0.001** | 3.425(2.402-4.883) | | **<0.001** | 2.466(1.643-3.702) |
| Intravenous line usage during med care | | |  |  |  | |  |
| No | 11(12.9%) | 74(87.1%) | 0.000 |  | |  |  |
| Yes | 162(20.7%) | 620(79.3%) | 0.092 | 1.758(0.912-3.389) | | **0.029** | 2.194(1.083-4.444) |
| Drainage tube usage during med care | | |  |  |  | |  |
| No | 163(19.5%) | 674(80.5%) | 0.000 |  | |  |  |
| Yes | 10(33.3%) | 20(66.7%) | 0.067 | 2.067(0.95-4.502) | | **0.033** | 2.449(1.074-5.587) |
| NG tube usage during med care | | |  |  |  | |  |
| No | 159(19.3%) | 664(80.7%) | 0.000 |  | | 0.000 |  |
| Yes | 14(31.8%) | 30(68.2%) | **0.047** | 1.949(1.01-3.761) | | 0.099 | 1.77(0.899-3.488) |
| Having chronic illness | |  |  |  | |  |  |
| No | 100(19.1%) | 423(80.9%) | 0.000 |  | |  |  |
| Yes | 115(11.9%) | 848(88.1%) | **<0.001** | 0.574(0.428-0.768) | | **0.041** | 1.646(1.021-2.655) |
| Having chronic wound infection on the skin | | |  |  |  | |  |
| No | 108(11.5%) | 833(88.5%) | 0.000 |  | |  |  |
| Yes | 7(31.8%) | 15(68.2%) | **0.006** | 3.599(1.435-9.025) | | 0.188 | 2.051(0.703-5.98) |
| Having chronic urinary tract infection | | |  |  |  | |  |
| No | 99(10.7%) | 825(89.3%) | 0.000 |  | |  |  |
| Yes | 16(41%) | 23(59%) | **<0.001** | 5.797(2.963-11.34) | | **<0.001** | 5.59(2.633-11.872) |
| Having chronic respiratory tract infection | | |  |  |  | |  |
| No | 98(11.2%) | 775(88.8%) | 0.000 |  | |  |  |
| Yes | 17(18.9%) | 73(81.1%) | **0.035** | 1.842(1.043-3.25) | | **0.027** | 2.146(1.092-4.217) |
| For neonates only | |  |  |  | |  |  |
| The educational level of family | | |  |  |  | |  |
| Illiterate | 9(29%) | 22(71%) | 0.243 | 0.468(0.13-1.676) | | 0.652 | 0.724(0.178-2.942) |
| Read and write | 8(11.9%) | 59(88.1%) | **0.004** | 0.155(0.044-0.543) | | **0.014** | 0.188(0.049-0.714) |
| Primary school | 16(28.1%) | 41(71.9%) | 0.175 | 0.446(0.139-1.433) | | 0.404 | 0.58(0.161-2.084) |
| Second school | 34(56.7%) | 26(43.3%) | 0.488 | 1.495(0.48-4.653) | | 0.424 | 1.647(0.484-5.604) |
| Higher Educa | 7(46.7%) | 8(53.3%) | 0.796 |  | | 0.000 |  |
| History of neonatal incubation | |  |  |  | |  |  |
| No | 10(16.1%) | 52(83.9%) | 0.000 |  | |  |  |
| Yes | 64(38.1%) | 104(61.9%) | **0.002** | 3.2(1.519-6.74) | | **0.005** | 3.186(1.419-7.151) |
| Neonatal mode of delivery | | |  |  |  | |  |
| NVD | 46(27.7%) | 120(72.3%) | 0.000 |  | |  |  |
| C/S | 28(43.8%) | 36(56.3%) | **0.021** | 2.029(1.114-3.695) | | 0.46 | 1.322(0.63-2.776) |
| Premature rupture of the membrane during delivery | | | |  |  | |  |
| >18 hours | 8(17.8%) | 37(82.2%) | 0.010 |  | | 0.120 |  |
| <18 hours | 52(32.7%) | 107(67.3%) | 0.057 | 2.248(0.977-5.17) | | 0.249 | 1.691(0.692-4.135) |
| No rupture(C/S) | 14(53.8%) | 12(46.2%) | **0.002** | 5.396(1.82-15.978) | | **0.04** | 3.725(1.064-13.043) |

Wards and their department = B6, NICU; C/W, causality; B4, SICU, MICU/PMICU; D7, Pediatrics Hematology; EOPD Emergency outpatient department; B7, pediatrics medical & surgery; C7, Pediatrics Medical; D8, Medical; B5, Medical & Neurosurgery; Values in bold are statistically significant.
